# Long-Term Structural Health Monitoring System for a High-Speed Railway Bridge Structure

**DOI:** 10.1155/2015/250562

**Published:** 2015-09-14

**Authors:** You-Liang Ding, Gao-Xin Wang, Peng Sun, Lai-Yi Wu, Qing Yue

**Affiliations:** ^1^Key Laboratory of Concrete and Prestressed Concrete Structures of Ministry of Education, Southeast University, Nanjing 210096, China; ^2^Department of Civil and Environmental Engineering, Rice University, Houston, TX 77005, USA; ^3^China Railway Major Bridge (Nanjing) Bridge and Tunnel Inspect & Retrofit Co., Ltd., Nanjing 210032, China

## Abstract

Nanjing Dashengguan Bridge, which serves as the shared corridor crossing Yangtze River for both Beijing-Shanghai high-speed railway and Shanghai-Wuhan-Chengdu railway, is the first 6-track high-speed railway bridge with the longest span throughout the world. In order to ensure safety and detect the performance deterioration during the long-time service of the bridge, a Structural Health Monitoring (SHM) system has been implemented on this bridge by the application of modern techniques in sensing, testing, computing, and network communication. The SHM system includes various sensors as well as corresponding data acquisition and transmission equipment for automatic data collection. Furthermore, an evaluation system of structural safety has been developed for the real-time condition assessment of this bridge. The mathematical correlation models describing the overall structural behavior of the bridge can be obtained with the support of the health monitoring system, which includes cross-correlation models for accelerations, correlation models between temperature and static strains of steel truss arch, and correlation models between temperature and longitudinal displacements of piers. Some evaluation results using the mean value control chart based on mathematical correlation models are presented in this paper to show the effectiveness of this SHM system in detecting the bridge's abnormal behaviors under the varying environmental conditions such as high-speed trains and environmental temperature.

## 1. Introduction

The environmental actions (wind, temperature, rain, earthquake, etc.) and the bridge usage (traffic loading) can continually modify the behavior and cause deterioration of long-span bridges in their long-term lifespan. Over the past several decades, a significant research effort has focused on the development of long-term Structural Health Monitoring (SHM) systems [1, 2]. Generally, the objective of the SHM system is to gather various reliable information that can be used to detect and follow the evolution of the bridge's condition state, locate and quantify damage, and perform reliability-based assessment so as to give the engineer or administrant a great variety of options with respect to maintenance intervention. In China, governments, as well as scientists and engineers, pay much attention to the long-term health monitoring of long-span highway bridges. Typical SHM implementations in highway bridges include Tsing Ma Bridge [3], Kap Shui Mun Bridge [3], Ting Kau Bridge [3], Stonecutters Bridge [3], Sutong Bridge [4], and Runyang Bridge [5]. The modular concept used in the design of the SHM systems for Tsing Ma Bridge, Kap Shui Mun Bridge, and Ting Kau Bridge has a significant influence on the design of new SHM systems in Hong Kong and China. The six integrated modules are the sensory system, the data acquisition and transmission system, the data processing and control system, the structural health evaluation system, the structural health data management system, and the inspection and maintenance system. A SHM system for long-span bridges should be able to monitor the loading and structural parameters set by the bridge designer so that the bridge performance under current and future loading conditions can be evaluated.

With the recent development of design theories and construction techniques for high-speed railway in China, many high-speed railway bridges have been built during the last few years. Maintaining the safety of these high-speed railway bridges is crucial to the safety of passengers and trains. Therefore, research efforts should be focused on the SHM systems of high-speed railway bridges. This paper reports the development and implementation of a SHM system of Nanjing Dashengguan Bridge. Nanjing Dashengguan Bridge, which serves as the shared corridor crossing Yangtze River for both Beijing-Shanghai high-speed railway and Shanghai-Wuhan-Chengdu railway, is the first 6-track high-speed railway bridge with the longest span throughout the world. It is the largest bridge with the heaviest design loading of all the high-speed railway bridges by far, because its main span is 336 m and its railway contains 6 tracks. Also the design speed of 300 km/h of the bridge is on the advanced level in the world. Due to these remarkable characteristics including long span of the main girder, heavy design loading, and high speed of trains, it is necessary to monitor the real-time health status of the bridge during its service period. With the state-of-the-art techniques of current monitoring systems of highway bridges, the health monitoring system for this bridge was designed to be concise, practical, reliable, and cost efficient. The main purpose of this system is to monitor the bridge's behaviors under the varying environmental conditions and detect the bridge's abnormal behaviors which may threaten the structural safety. This paper introduces the deployment and functions of this system including a sensor system, a data acquisition and transmission system, a data management system, and a structural evaluation system. Some evaluation results of the bridge subjected to varying environmental conditions such as high-speed trains and environmental temperature are also presented.

## 2. Structural Health Monitoring System

**Figure 1:**
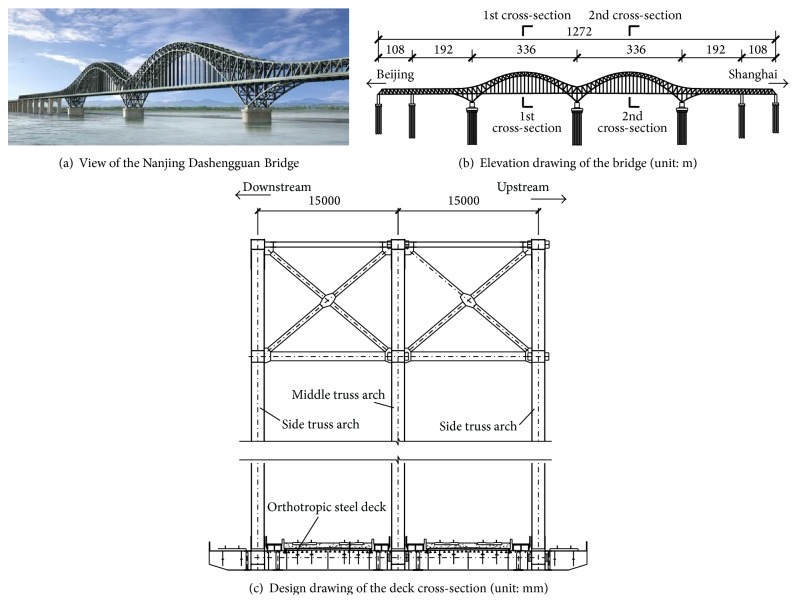
Nanjing Dashengguan Bridge.

Nanjing Dashengguan Bridge shown in Figure 1(a) is a steel truss arched bridge with the span arrangement (108 + 192 + 2 × 336 + 192 + 108) m, which supports a six-line railway, including two regular rails, two high-speed railways, and two subway lines. The elevation drawing of the bridge and the deck cross-section are shown in Figures 1(b) and 1(c), respectively. The three truss planes in the longitudinal direction were used with a spacing of 15 m, which was selected to provide the large stiffness for such heavy live loads. The use of a steel box as the deck system is another feature in the design of this steel truss arc, which is adopted as the bottom chord of the entire truss segment to reduce uneven deflection on the deck. The bearing system of the bridge is that the 7# pier is linked to the bridge deck so that the bridge deck is restricted from moving in the longitudinal direction at the 7# pier. And there have no longitudinal restraints between the bridge deck and other piers. Due to the remarkable characteristics of Nanjing Dashengguan Bridge including long span of the main girder, heavy design loading, and high speed of trains, a long-term SHM system was designed and installed on the Nanjing Dashengguan Bridge shortly after it was opened to railway traffic. This online SHM system was designed to be a concise monitoring system with a minimum of 28 sensors to monitor the key parameters. The entire system consists of a sensor system, a data acquisition system, a data transmission system, a data management system, and a structural evaluation system. The primary purpose of the system is to monitor in-service performance of the bridge structure under the varying environmental conditions such as high-speed trains and environmental temperature and to provide early warning of abnormal changes in in-service performance of the bridge that might be unsafe to bridge structure. The procedure of designing and implementing the SHM system includes three major stages as follows: (1) the stage of planning and design includes the consideration of the physical quantities to be measured, the selection of sensors appropriate for measuring such physical parameters, and the optimal placement of the sensors. (2) The stage of installation and calibration includes a series of static and dynamic experiments performed to check the operation of the monitoring system after the completion of the bridge. (3) The stage of main operation includes the test operation of the monitoring system, training of the maintenance crew, and the replacement of malfunctioning sensing devices.

The design objective of the SHM system is to monitor the structural health under daily service condition. With regard to the bridge site of Nanjing Dashengguan Bridge, there are very low probability events of Typhoon and earthquake. Thus, the train loading and temperature action are the most important factors that affect the daily performance of the bridge. Due to the design requirements, the structural responses of the bridge under train loading are small and conservative. However, the axial movements and thermal stresses of the bridge under temperature action are considerable due to the long span of the bridge. For instance, the maximum thermal strain and train-induced strain in the steel truss arch are about 250 *με* and 50 *με*, respectively. For bridge condition assessment it is of paramount importance to characterize normal variability of structural parameters due to temperature effects. Therefore, in order to control the overall budget of the SHM system without losing major information on structural daily behavior, the structural parameters to be monitored in the Nanjing Dashengguan Bridge were determined using the structural sensitivity analysis with the finite element model under the designed temperature action, as shown in Table 1. Other important parameters including accelerations of steel deck and fatigue of steel deck were also monitored. Considering that the displacement amplitudes of steel deck under the train loading can be derived from the acceleration of steel deck, the vertical displacements of steel deck were not chosen to monitor.

### 2.1. Temperature and Static Strain Monitoring of Truss Arch

**Figure 2:**
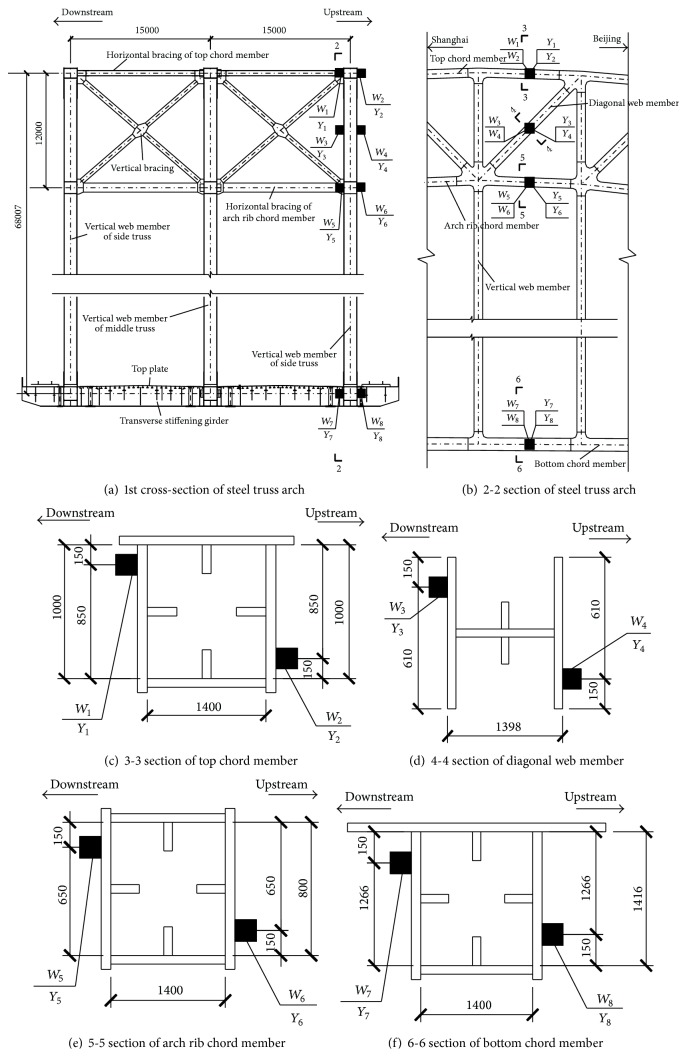
Layout of temperature and static strain gauges on the steel truss arch (unit: mm).

As shown in Figure 1(b), temperature and static strain monitoring of truss arch is performed at the 1st cross-section in the middle of the first main span of the bridge. Eight FBG temperature and strain gauges are both installed on the 1st cross-section to simultaneously measure temperature field data and static strain field data in the steel truss arch as shown in Figure 2. It can be seen from Figures 2(c)~2(f) that the sensors were installed at the top chord member, diagonal web member, arch rib chord member, and bottom chord member of the truss arch (where *W*_*i*_ denotes *i*th temperature sensor, *i* = 1,2,…, 8. *Y*_*i*_ denotes *i*th strain sensor, *i* = 1,2,…, 8). The specification of FBG temperature sensors and strain gauges is FBG4700 and FBG4100 supplied by China Geokon Instruments Co., Ltd. Sampling frequency of temperature and static strain data collection is set to 1 Hz.

### 2.2. Dynamic Strain Monitoring of Steel Deck

**Figure 3:**
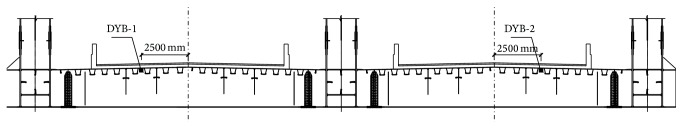
Layout of dynamic strain gauges on the steel deck.

Dynamic strain gauges are installed at the 1st cross-section in the middle of the first main span of the bridge so as to monitor the fatigue performance of orthotropic steel deck as shown in Figure 3. It can be seen that the dynamic strain gauges DYB-1 and DYB-2 monitor the long-term fatigue effects of deck-rib welded detail induced by the traveling high-speed trains. The specification of dynamic strain gauges is FBG4100 supplied by China Geokon Instruments Co., Ltd. Sampling frequency of dynamic strain data collections is set to 50 Hz.

### 2.3. Acceleration Monitoring of Steel Deck

**Figure 4:**
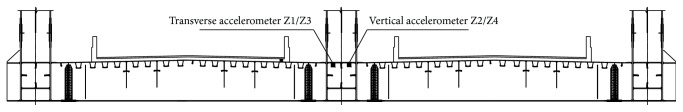
Layout of accelerometers on the steel deck.

In order to monitor the transverse and vertical acceleration responses on the two main spans induced by the traveling high-speed trains, one transverse accelerometer and one vertical accelerometer were installed at the 1st cross-section and 2nd cross-section in the middle of two main spans, respectively, as shown in Figure 4 (where *Z*_*i*_ denotes *i*th accelerometer, *i* = 1,2,…, 4). The specification of accelerometers is DH610 supplied by Donghua Testing Technology Co., Ltd. Sampling frequency of acceleration data collections is set to 200 Hz.

### 2.4. Longitudinal Displacement Monitoring of Piers

**Figure 5:**
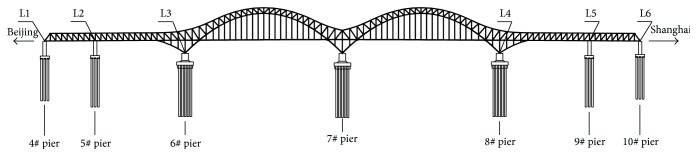
Layout of displacement transducers on the pier.

In the design of Nanjing Dashengguan Bridge, the 7# pier is linked to the bridge deck so that the bridge deck is restricted from moving in the longitudinal direction at the 7# pier. And there have no longitudinal restraints between the bridge deck and other piers. Therefore, 6 displacement transducers are located on the piers and used for the measurement of longitudinal displacements at the 4# pier, 5# pier, 6# pier, 8# pier, 9# pier, and 10# pier as shown in Figure 5 (where *L*_*i*_ denotes *i*th displacement transducer, *i* = 1,2,…, 6). The specification of displacement transducers is CLMD2 supplied by ASM's in Germany. Finally, it should be noted that the DAQs of the SHM system are located in the 6# and 8# piers.

## 3. Evaluation of Structural Condition Using Long-Term Monitoring Data

Using long-term monitoring data collected from the SHM system of Nanjing Dashengguan Bridge, structural condition assessment involves four aspects: evaluation of vibration performance of the main girder, evaluation of static performance of steel truss arch, evaluation of movement performance of pier, and evaluation of fatigue performance of steel deck.

### 3.1. Evaluation of Vibration Performance of the Main Girder

The main function for the SHM system is to detect the performance degradation of structures as early as possible and to provide essential references for maintenance of actual bridges. For Nanjing Dashengguan Bridge, the vibration and strain of the main girder together with the longitudinal displacement of piers are three important items to monitor. Three kinds of performance evaluation are conducted accordingly. Those are evaluation of vibration performance of the main girder, evaluation of static performance of steel truss arch, and evaluation of movement performance of pier. Firstly, degradation or damage of local members may occur with the increase of service period, which will reduce the structural stiffness of bridge. Thus, the dynamic property of bridge structure is affected significantly by the structural stiffness reduction and will cause unusual variations of the structural vibration. Such unusual behavior will affect running safety of the train directly. Secondly, damage of local members may induce redistribution of the internal forces in the whole bridge under the temperature action. The reason is that Nanjing Dashengguan Bridge belongs to statically indeterminate structure. Therefore, static strain monitoring is necessary for typical members under temperature actions. Thirdly, the longitudinal displacement behavior of the bridge pier is crucial for running safety of the train under the temperature action. Once the abnormal variation of the item is detected, inspection may be evolved timely for detection of the bridge piers. In this section, the evaluation of vibration performance of main girder is firstly discussed.

For high-speed railway bridges, vibration performance of the main girder subjected to the high-speed trains is important for passenger comfort and traffic safety [6, 7]. For instance, Zeng et al. [8] point out that the essential reason of train derailment is that the transverse vibration of the train-railway-bridge system becomes unstable. Hence, it is necessary to present a strategy for early warning of deterioration of vibration performance of the main girder using long-term health monitoring data.

**Figure 6:**
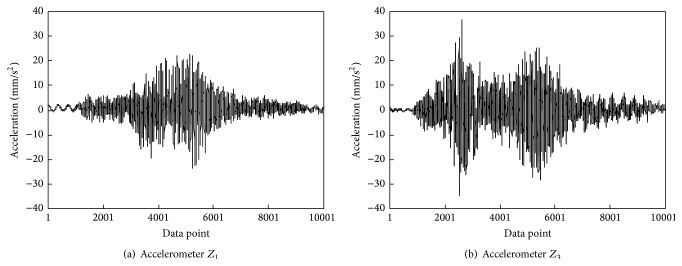
Transverse acceleration time histories of the main girder induced by a high-speed train.

**Figure 7:**
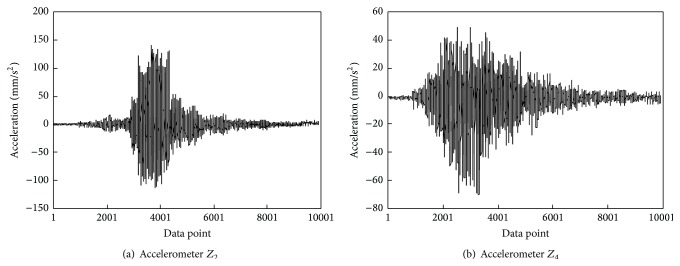
Vertical acceleration time histories of the main girder caused by a high-speed train.

Figures 6 and 7 illustrate the typical transverse and vertical acceleration time histories of the main girder measured from accelerometers* Z*_1_~*Z*_4_, respectively. It can be observed that when high-speed train passed the bridge, the transverse accelerations in the middle of the first span are much smaller than that of the second span and the vertical accelerations in the middle of the first span are much larger than that of the second span. These indicate that although the structural layouts of two main spans of the main girder are the same, there exists significant difference between the transverse and vertical vibration characteristics of two main spans due to the rail irregularity in transverse and vertical directions. Thus, there is a need to monitor the transverse and vertical accelerations of two main spans in the long term so as to realize anomaly alarms for vibration performance of the main girder.

**Figure 8:**
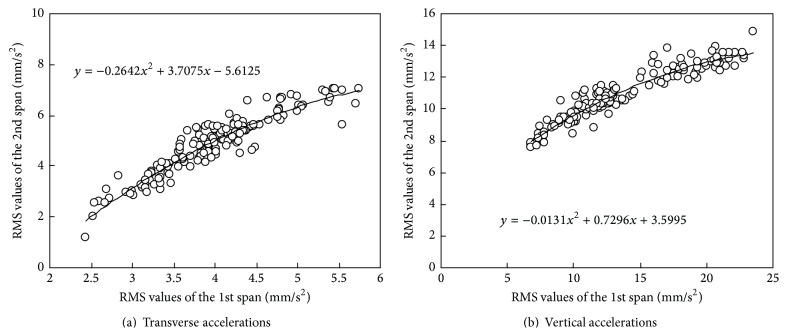
Cross-correlations between RMS values of accelerations measured in the middle of two main spans.

Hence, in the present study, the RMS values of the accelerations are utilized as the monitoring parameters to represent the transverse and vertical vibration characteristic of the main girder. The cross-correlation between the RMS values of the accelerations in the middle of two main spans is further investigated when the high-speed trains pass through the bridge.  Figure 8 shows the scatter plots between the RMS values of the accelerations and shows the fitted results by using quadratic polynomial as well.

**Figure 9:**
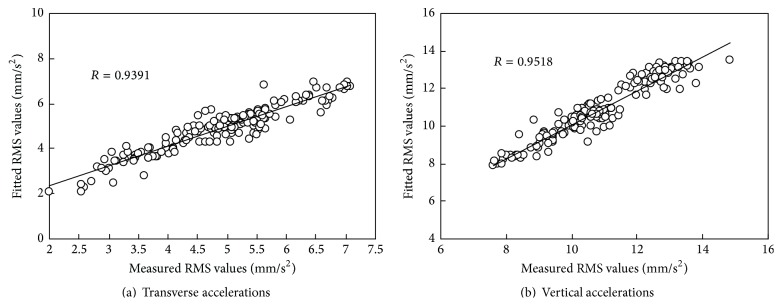
Fitting effect of cross-correlation between RMS values of accelerations by using quadratic polynomial.

**Figure 10:**
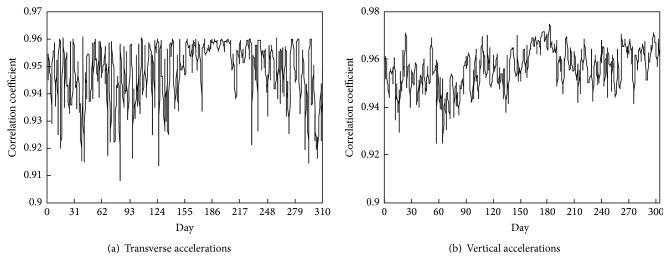
Daily computed correlation coefficients in 2013.

By using the fitting formula constructed in Figure 8, scatter plot between the RMS values of the accelerations in the middle of the second span and the corresponding fitted values is drawn in Figure 9, where the fitted values were obtained by taking the RMS values of the accelerations in the middle of the first span into the fitting formula of the quadratic polynomial. The correlation coefficient of the measured RMS values and fitted values on February 1st, 2013, is 0.9391 and 0.918 for transverse and vertical accelerations, respectively, as shown in Figure 9. Computed using the same method, all the daily correlation coefficients in 2013 are more than 0.90 for transverse and vertical accelerations, as shown in Figure 10.

**Figure 11:**
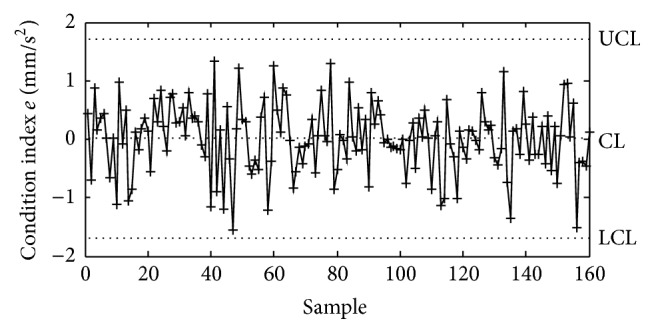
Mean value control chart for accelerations in the middle of the second span.

The analysis results indicate that good cross-correlation exists between the RMS values of the transverse and vertical accelerations on the two main spans. Multivariable control chart is used here to monitor the measured changes in the transverse and vertical accelerations caused by deterioration of the vibration performance. Firstly, the condition index *e* for early warning of abnormal vibration behavior is defined as the difference between the measured and predicted RMS values of the accelerations in the middle of the second span:

(1) $$\begin{array}{c} e={\text{RMS}}_{m}-{\text{RMS}}_{p}, \end{array}$$

where RMS_*m*_ is the measured RMS values of the accelerations in the middle of the second span; RMS_*p*_ is the predicted RMS values of the accelerations in the middle of the second span, which is obtained by taking the RMS values of the accelerations in the middle of the first span into the fitting formula of the quadratic polynomial in the healthy condition. Then a mean value control chart is employed to monitor the time series of *e* with regard to both transverse and vertical accelerations. The time series of *e*, taken when the structural vibration is in good condition, will have some distribution with mean *μ* and variance *σ* ^2^. If the mean and standard deviation are known, a control chart is constructed by drawing a horizontal line CL at *μ* and two more horizontal lines representing the upper and lower control limits. The upper limit UCL is drawn at *μ* + *kσ* and the lower limit LCL at *μ* − *kσ*. For online monitoring, the controlling parameter *k* is chosen so that when the structural vibration is in good condition all observation samples fall between the control limits. When the new measurement is made, the structural abnormal vibration condition can be detected if an unusual number of samples fall beyond the control limits.  Figure 11 shows the mean value control chart using the measured and predicted RMS values of the accelerations shown in Figure 9. The upper and lower limits are determined using the controlling parameter *k* = 2.612. Hence, a long-term monitoring on the cross-correlation model of RMS values of the transverse and vertical accelerations can realize the early warning of deterioration of the vibration performance.

### 3.2. Evaluation of Static Performance of Steel Truss Arch

#### 3.2.1. Data of Temperature Field and Static Strains

**Figure 12:**
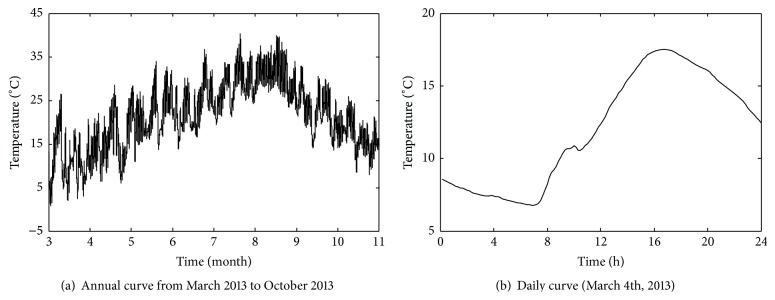
Time-history curves of temperature data *T*_1_.

**Figure 13:**
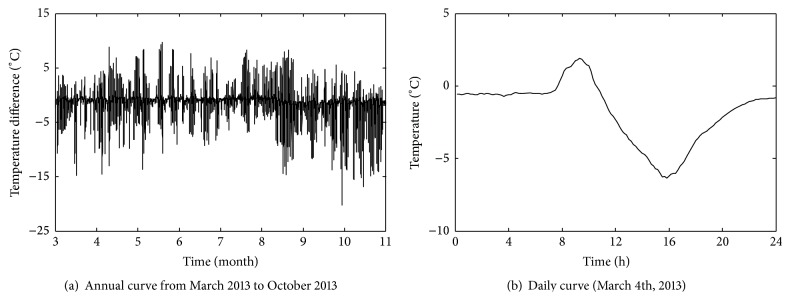
Time-history curves of temperature difference data *T*_12_.

**Figure 14:**
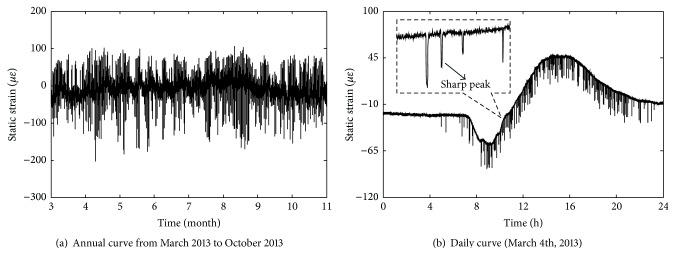
Time-history curves of static strain data *S*_1_.

Temperature data from temperature sensor *W*_*i*_ is denoted by *T*_*i*_, temperature difference data acquired by *T*_*i*_ minus *T*_*j*_ is denoted by *T*_*ij*_ (*T*_*ij*_ = *T*_*i*_ − *T*_*j*_), and static strain data from strain sensor *Y*_*i*_ is denoted by *S*_*i*_ (*i*, *j* = 1,2,…, 8). As for illustration, the time-history curves of annual change and daily change in temperature *T*_1_ and temperature difference *T*_12_ are shown in Figures 12 and 13, respectively. And the time-history curves of annual change and daily change in static strain data *S*_1_ are shown in Figure 14 (negative values denote compressive static strain and positive values denote tension static strain). From Figure 14, it can be seen that time-history curve of *S*_1_ contains many sharp peaks caused by high-speed trains, with each peak corresponding to the time when one train passes through the bridge. And the static strains caused by high-speed trains are obviously lower than that of temperature field including temperature data and temperature difference data.

**Figure 15:**
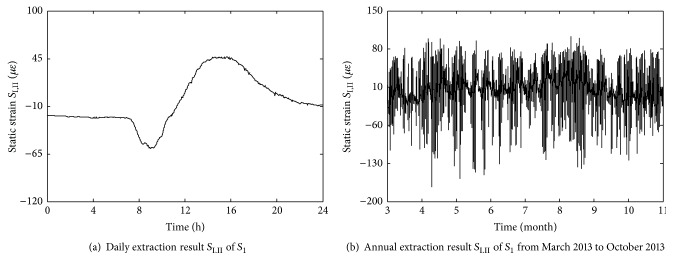
Extraction results *S*_I,II_ of static strain data *S*_1_.

As shown in Figure 14, static strain data in steel truss arch mainly contains three parts: static strain *S*_I_ caused by temperature, static strain *S*_II_ caused by temperature difference, and static strain *S*_III_ caused by train. In order to construct the correlation between temperature field and static strain in steel truss arch, static strain *S*_I_ and static strain *S*_II_ (denoted by *S*_I,II_) must be extracted from the static strain data. In the present study, wavelet packet decomposition method is used to extract *S*_I,II_ considering the high-frequency characteristics of static strain data caused by trains. By using the wavelet packet decomposition, static strain data can be decomposed scale by scale into different frequency bands, and each decomposition coefficient corresponds to its frequency band [9, 10]. Each decomposition coefficient can be reconstructed into time-domain signals with constraints of its own frequency band. Taking the daily time-history curve of static strain data *S*_1_ shown in Figure 14(b) as an example, it is decomposed within 8 scales and the decomposition coefficient *C*_*j*_^0,8^ is specially selected and then reconstructed as *S*_I,II_ shown in Figure 15(a). It can be seen that sharp peaks caused by trains are removed effectively and the static strains which are caused by temperature field are retained as well. Furthermore, the annual extraction result *S*_I,II_ of *S*_1_ from March 2013 to October 2013 is shown in Figure 15(b).

#### 3.2.2. Correlation Model between Temperature Field and Static Strains

In constructing the correlation model between temperature field and static strains, data from March 2013 to October 2013 are chosen as training data and data in November 2013 are chosen as test data for verification of the correlation model. The static strain *S*_I,II_ is expressed as follows:

(2) $$\begin{array}{c} S_{\text{I,II}}=\overset{8}{\underset{i=1}{\sum }}\alpha_{i}T_{i}+\overset{16}{\underset{j=1}{\sum }}\beta_{j}D_{j}+c, \end{array}$$

where *T*_*i*_ is *i*th temperature data from set {*T*_1_, *T*_2_, *T*_3_, *T*_4_, *T*_5_, *T*_6_, *T*_7_, *T*_8_}, *D*_*j*_ is *j*th temperature difference data from set {*T*_12_, *T*_34_, *T*_56_, *T*_78_, *T*_13_, *T*_15_, *T*_17_, *T*_35_, *T*_37_, *T*_57_, *T*_24_, *T*_26_, *T*_28_, *T*_46_, *T*_48_, *T*_68_}, *α*_*i*_ is *i*th linear expansion coefficient of *T*_*i*_, *β*_*j*_ is *j*th linear expansion coefficient of *T*_*ij*_, and *c* is the constant term.

Considering that total 24 linear expansion coefficients and one constant term in (2) are very complicated for calculation, principal component analysis is further used to simplify (2), which aims at finding small amounts of comprehensive factors to represent main information of temperature and temperature difference data. By using principal component analysis, the comprehensive factors can be found by transforming temperature and temperature difference data into factor component space [11, 12]. For temperature data the explained variance of the first potential comprehensive factor *P*_1_ reaches 96.7%, apparently larger than the other potential comprehensive factors. Thus, *P*_1_ is selected as the comprehensive factor. Meanwhile, for temperature difference data using principal component analysis, the explained variance of the first three potential comprehensive factors *R*_1_, *R*_2_, and *R*_3_ reaches 98.8%, which are selected as the comprehensive factors.

Therefore, the correlation models between *S*_I,II_ and temperature field can be expressed as follows:

(3) $$\begin{array}{c} S_{\text{I,II}}=\lambda_{1}\left(\;{\left[\;E\;\right]}_{1\times 8}{\overset{⃑}{T}}_{8\times 1}\;\right)+{\overset{⃑}{\gamma }}_{1\times 3}\left(\;{\left[\;F\;\right]}_{3\times 16}{\overset{⃑}{D}}_{16\times 1}\;\right)+c, \end{array}$$

where ${\overset{⃑}{T}}_{8\times 1}$ denotes one vector containing eight temperature data; ${\overset{⃑}{D}}_{16\times 1}$ denotes one vector containing sixteen temperature difference data *D*_*j*_ (*j* = 1,2,…, 16); [*E*]_1×8_ and [*F*]_3×16_ denote transformation matrix, which can be calculated using principal component analysis [11, 12]; *λ*_1_ is performance parameter of first potential comprehensive factor *P*_1_; ${\overset{⃑}{\gamma }}_{1\times 3}=[\gamma_{1},\gamma_{2},\gamma_{3}]$ is one vector containing three performance parameters corresponding to first three potential comprehensive factors *R*_1_, *R*_2_, and *R*_3_. The values of performance parameters *λ*_1_, ${\overset{⃑}{\gamma }}_{1\times 3}$, and *c* are estimated using training data by multivariate linear regression method [13] and are shown in Table 2.

**Figure 16:**
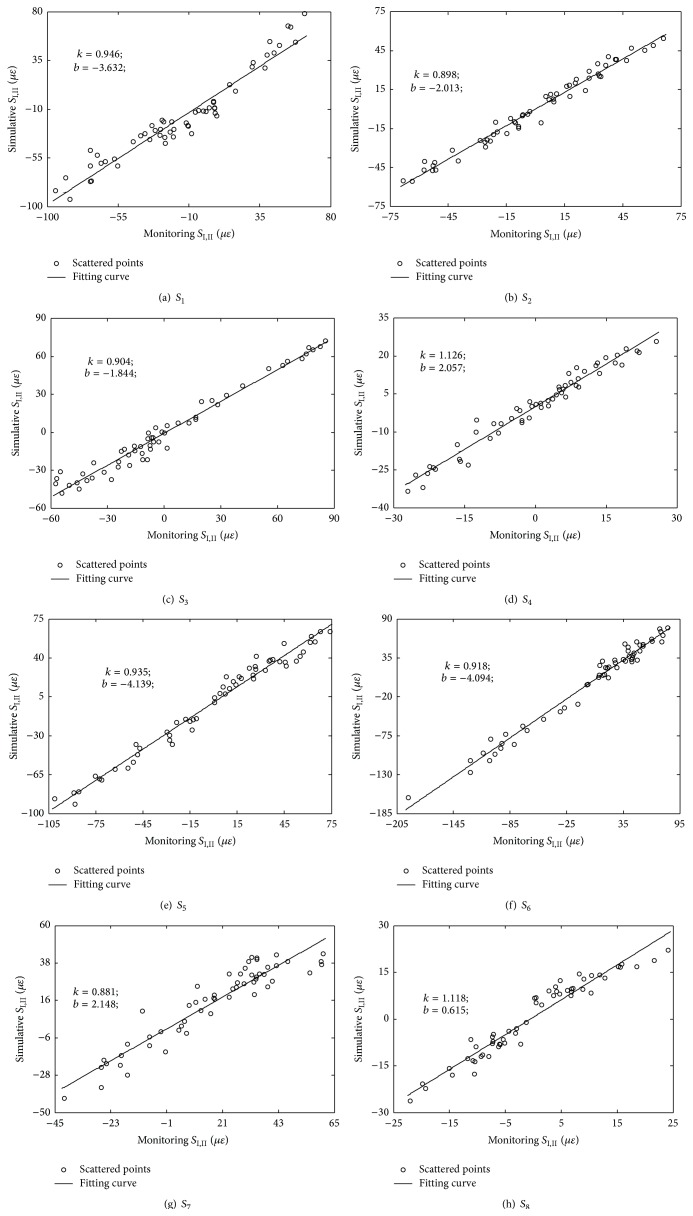
Correlation between simulative *S*_I,II_ and monitoring *S*_I,II_.

**Figure 17:**
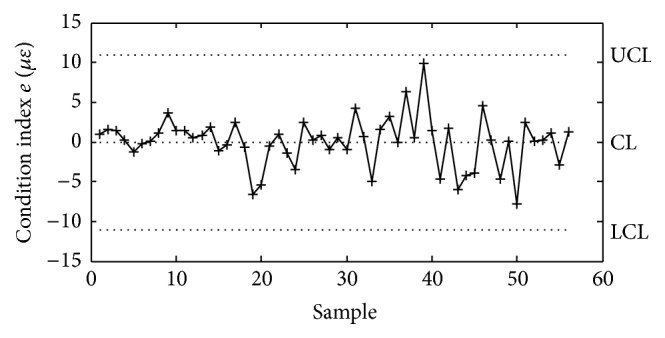
Mean value control chart for static strains of the steel truss arch.

In order to verify the effectiveness of the correlation models shown in (2), test data in November 2013 are used to obtain the simulative *S*_I,II_. The scattered points between simulative *S*_I,II_ and monitoring *S*_I,II_ are plotted as shown in Figure 16, which are further fitted by linear function:

(4) $$\begin{array}{c} S_{s}=kS_{m}+b, \end{array}$$

where *S*_*s*_ denotes simulative *S*_I,II_, *S*_*m*_ denotes monitoring *S*_I,II_, and *k*, *b* are fitting parameters, with the fitting results shown in Figure 16 as well. The fitting results verify the effectiveness of the correlation models. A multivariable mean value control chart is also employed to monitor the measured abnormal changes in the static strains of the steel truss arch. The condition index *e* for early warning of deterioration of the static performance of steel truss arch is defined as the difference between the measured and predicted static strains:

(5) $$\begin{array}{c} e=S_{m}-S_{p}, \end{array}$$

where *S*_*m*_ is the measured static strain *S*_I,II_; *S*_*p*_ is the predicted static strain *S*_I,II_, which is obtained by using the correlation models shown in (2) in the healthy condition.  Figure 17 shows the mean value control chart for static strains of the steel truss arch using measurement data in November 2013. The upper and lower limits are determined using the controlling parameter *k* = 2.235. Hence, a long-term monitoring on the correlation models between temperature field and static strains is suitable for early warning of deterioration of the static performance of steel truss arch.

### 3.3. Evaluation of Movement Performance of Pier

**Figure 18:**
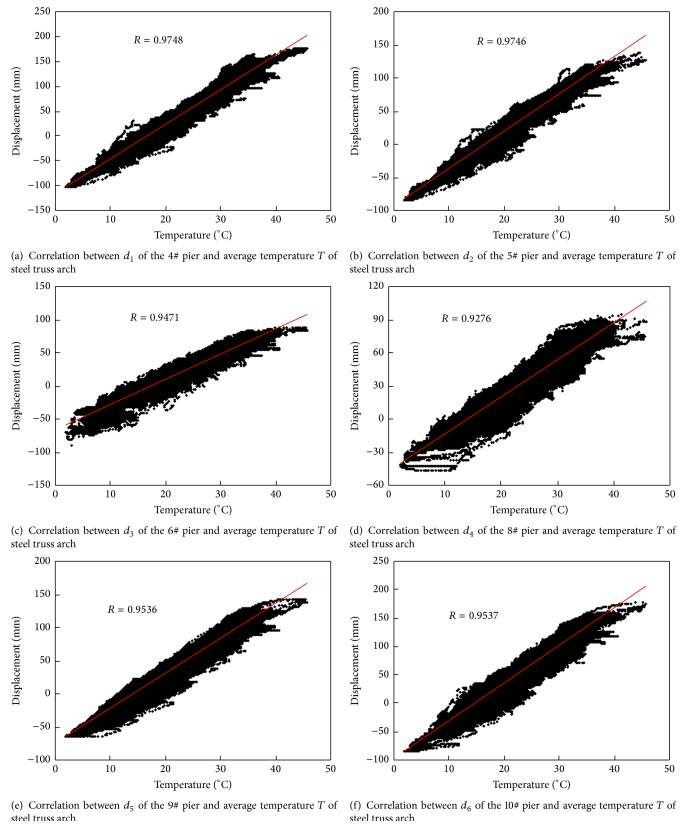
Correlation between longitudinal displacement and average temperature.

Longitudinal displacements of piers and temperature field data in steel truss arch collected from March 2013 to October 2013 are used for evaluation of movement performance of piers. Longitudinal displacement data from sensors *L*_*i*_ (*i* = 1,2,…, 6) are denoted by *d*_*i*_(*t*) (where *t* means time), respectively. Meanwhile, temperature field data from sensors *W*_*i*_ (*i* = 1,2,…, 8) are denoted by *T*_*i*_(*t*), respectively, and their averaged value *T*(*t*) is used to represent the average temperature of the steel truss arch. For simplicity, taking 10 minutes as basic time interval, the 10 min average displacements and temperatures are calculated and used as the representative values of this time interval. By this way, the displacements and temperatures in one day are reduced to 144 representative samples. Furthermore, the temperature-displacement scatter diagrams are plotted in Figure 18. An overall increase in longitudinal displacements of piers is observed with an increase in the average temperature of the bridge. And the correlation coefficients of the longitudinal displacements of piers and average temperature of the steel truss arch are all larger than 0.92, as shown in Figure 18.

A linear regression analysis between the 10 min average displacement *d*_*i*_(*t*) and the temperature *T*(*t*) is further performed to model the temperature-displacement correlations using the following equation [14]:

(6) $$\begin{array}{c} d_{i}\left(\;t\;\right)=\beta_{0}+\beta_{1}T\left(\;t\;\right), \end{array}$$

where the regression coefficients *β*_1_ and *β*_0_ were obtained by the least-squares method as

(7) β1=SdiTSTT,β0=d−i−β1T−,

where *S*_*d*_*i*_*T*_ is the covariance between the displacement and the temperature sequences; *S*_*TT*_ is the variance of the measured temperature sequences; and $\overset{-}{T}$ and ${\overset{-}{d}}_{i}$ are the means of the measured temperature and displacement sequences, respectively.  Table 3 summarizes the expressions of linear regression models of the displacement versus temperature.

**Figure 19:**
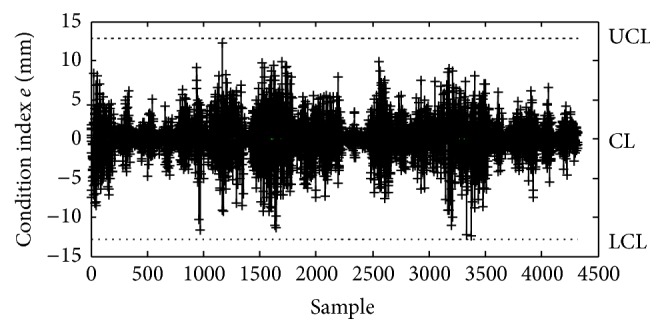
Mean value control chart for longitudinal displacements of piers.

The analysis results indicate that good correlation exists between the longitudinal displacements of piers and average temperature of steel truss arch. A multivariable mean value control chart is also employed to monitor the measured abnormal changes in the longitudinal displacements of piers. The condition index *e* for early warning of abnormal behavior of piers is defined as the difference between the measured and predicted longitudinal displacements:

(8) $$\begin{array}{c} e=d_{m}-d_{p}, \end{array}$$

where *d*_*m*_ is the measured displacement of the piers; *d*_*p*_ is the predicted displacement of the piers, which is obtained by using the linear regression models shown in Table 3 in the healthy condition.  Figure 19 shows the mean value control chart for longitudinal displacements of piers using measurement data in March 2013. The upper and lower limits are determined using the controlling parameter *k* = 2.804. Hence, a long-term monitoring on the temperature-displacement correlation model is suitable for real-time condition assessment for movement performance of piers.

### 3.4. Evaluation of Fatigue Performance of Steel Deck

Fatigue cracking is universal for orthotropic steel deck of highway bridges. Once the fatigue crack appears, extensive fatigue cracking may occur and repair is very difficult. Hence, infinite-fatigue-life design method is applied for Nanjing Dashengguan Bridge instead of finite-fatigue-life design method which is applied extensively in highway bridges. The infinite-fatigue-life design method is to control the fatigue stress amplitude in the range less than the value of fatigue cut-off limit refrained from fatigue cracking. Consequently, the purpose of equipping stain gauges in the SHM system of Dashengguan Bridge is to monitor the stress amplitude and the validity of fatigue design can be achieved by comparing the stress amplitude with fatigue stress limit.

**Figure 20:**
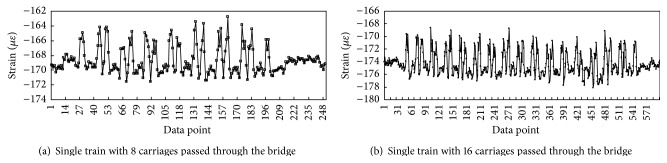
Typical strain time-history curves of DYB-2 when single train passed through the bridge.

The structural dynamic response is asymmetrical because of the irregularity of railway and braking force from the train even though the structure of Nanjing Dashengguan Bridge is spatially symmetrical. Hence, the stresses are not symmetrical measured by strain gauges from the upstream and downstream directions. However, the fatigue amplitudes of two strain gauges induced by trains are both extremely small. Thus, the strain history data of the strain gauge DYB-2 are merely selected in this paper to illustrate that the fatigue stress amplitude is much lower than the value of fatigue stress limit. The typical strain time-history curves of DYB-2 when single train with 8 carriages or 16 carriages passed through the bridge are shown in Figure 20, respectively. It can be seen that, during the passing of high-speed trains, both 8 carriages and 16 carriages have led to strain variations reaching to 2–8 *με*. And the number of strain peaks is 16 and 32 for trains with 8 carriages and 16 carriages, respectively, because the axle number of each carriage is 2.

**Figure 21:**
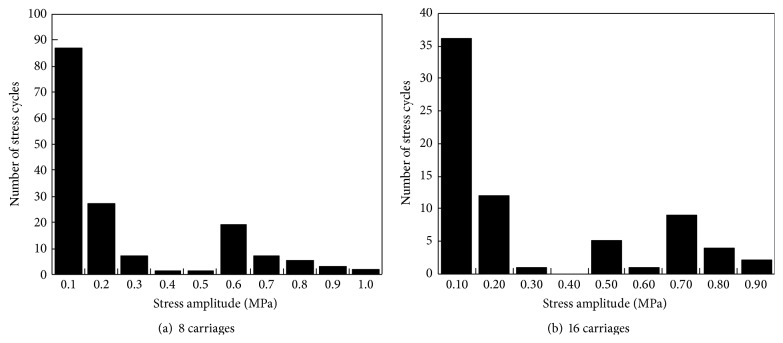
Spectra of stress amplitude calculated using the strain history data.

To perform fatigue evaluation, simplified rainflow cycle counting algorithm was firstly used to process strain history data and the spectrum of stress amplitude was obtained [15]. The spectra of stress amplitude calculated using the strain history data with regard to 8 carriages or 16 carriages are shown in Figure 21, respectively. It can be seen that the maximum stress amplitude is smaller than 1.0 MPa obtained from strain history curves under single train. Then according to Miner's law and standard of Eurocode 3, fatigue parameters of *S*_eq_ (equivalent stress amplitude) and *N*_*s*_ (stress cycle number) under single train were calculated as follows [16]:

(9) Seq=niSi5∑ni1/5,Ns=∑ni,

where *S*_*i*_ is *i*th stress amplitude, *n*_*i*_ is the number of stress cycles corresponding to *S*_*i*_, and 1/5 is slope value of log⁡*S*-log⁡*N* curve in the scope of lower stress amplitude according to the standard of Eurocode 3 [17].

Using (9), the equivalent stress amplitudes *S*_eq_ with regard to 8 carriages or 16 carriages are 0.75 MPa and 0.71 MPa, respectively. And the stress cycle numbers *N*_*s*_ are 22 and 45, respectively. Thus, the value of *S*_eq_ with regard to 8 carriages is similar to that of 16 carriages. However, the value of *N*_*s*_ with regard to 8 carriages is about twice as much as that of 16 carriages. Furthermore, as recommended by Eurocode 3 for rib-to-deck welded joint, the fatigue limit Δ*σ*_*L*_ is 29 MPa [17], and the value of *S*_eq_ when the high-speed train passes through bridge is far less than Δ*σ*_*L*_, which indicates that fatigue life of rib-to-deck welded joint in Nanjing Dashengguan Bridge can be considered infinite. Thus, the fatigue performance of steel deck satisfies the requirement of infinite-fatigue-life design method.

## 4. Conclusions

In the last few decades, Structural Health Monitoring (SHM) system has been widely installed in many highway bridges to continuously monitor the structural condition over an extended period of time. However, application of SHM system in high-speed railway bridges is far insufficient. This paper presents an analysis of the SHM system installed in Nanjing Dashengguan Bridge in China. The monitoring system is composed of a sensor system, a data acquisition and transmission system, a data management system, and a structural evaluation system. The monitoring system is currently in full operation, accumulating the bridge's behaviors under the varying environmental conditions such as high-speed trains and environmental temperature.

Based on long-term health monitoring data, general principles and analytical processes for structural evaluation are provided, and some evaluation results are reported. It can be shown from the analysis in this paper that the mathematical correlation models describing the overall structural behavior of the bridge can be obtained with the support of the health monitoring system, which includes cross-correlation models for accelerations, correlation models between temperature and static strains of steel truss arch, and correlation models between temperature and longitudinal displacements of piers. The mean value control chart is further employed to monitor the time series of differences between the measured structural parameters and the predicted parameters using these correlation models. The upper and lower control limits of the control chart are then determined and abnormal behaviors can be detected if the time series of differences fall beyond the control limits during the monitoring period, which can result in useful suggestions to bridge management authorities by reasonably evaluating the monitored data.

## Figures and Tables

**Table 1:** Sensitivity analysis of bridge parameters for SHM design.

Structure	Key position	Critical member	Parameter	Importance	Variation under temperature action	Sensitivity	Decision
Pier	Side spans	4# and 10# piers	Longitudinal displacement	Critical	229 mm	Sensitive	Monitor

Main girder	In the middle of the main span	Steel deck	Vertical displacement	Less-important	35 mm	Less-sensitive	No

Truss arch	In the middle of the main span	Top chord member, diagonal web member, arch rib chord member, and bottom chord member	Static strain	Critical	280 *με*	Sensitive	Monitor

Truss arch	In the middle of the main span	Top chord member and bottom chord member	Temperature	Critical	60°C	Sensitive	Monitor

Main girder	In the middle of the main span	Steel deck	Temperature	Less-important	60°C	Less-sensitive	No

Whole bridge	/	/	Frequency	Important	/	Nonsensitive	Monitor one

Truss arch	In the middle of the main span	Top chord member	Wind	Less-important	/	Less-sensitive	No

**Table 2:** Performance parameters *λ*_1_, *γ*_1_, *γ*_2_, *γ*_3_, and *C* in correlation models.

Static strain data	*λ*_1_	*γ*_1_	*γ*_2_	*γ*_3_	*C*
*S*_I,II_ of *S*_1_	0.454	−4.711	−2.597	−4.944	−46.806
*S*_I,II_ of *S*_2_	1.585	0.013	4.104	−1.247	−70.238
*S*_I,II_ of *S*_3_	0.301	3.416	−2.098	2.968	−6.884
*S*_I,II_ of *S*_4_	0.350	−0.734	0.351	0.948	−23.597
*S*_I,II_ of *S*_5_	0.179	−4.219	1.827	3.165	−22.049
*S*_I,II_ of *S*_6_	0.062	−3.245	8.242	−5.398	−22.862
*S*_I,II_ of *S*_7_	−0.367	2.411	−0.598	5.531	17.075
*S*_I,II_ of *S*_8_	0.611	2.188	−1.130	2.018	−32.799

**Table 3:** Summary of linear regression models.

Pier	Regression function
4# pier	*d*_1_ = −115.24 + 6.96*T*
5# pier	*d*_2_ = −92.53 + 5.62*T*
6# pier	*d*_3_ = −66.28 + 3.82*T*
8# pier	*d*_4_ = −46.58 + 3.36*T*
9# pier	*d*_5_ = −76.05 + 5.30*T*
10# pier	*d*_6_ = −98.50 + 6.64*T*
